# Is there a “weekend effect” in adenoma detection rate?A single-center retrospective study

**DOI:** 10.1371/journal.pone.0345613

**Published:** 2026-04-06

**Authors:** Baorang Cai, Bijuan Lin, Conghua Song, Zhizhong Lu, Xinxiang Huang, Dongsheng Chen, Dongdong Lv, Huifeng Wu, Lijuan Zheng, Yuxiang Wang, Chenxing Jian, Chao Zhong Huang

**Affiliations:** 1 Endoscopy Center, Affiliated Hospital of Putian University, China; 2 Department of Rehabilitation Medicine, Affiliated Hospital of Putian University, China; 3 Department of Anorectal Surgery, Affiliated Hospital of Putian University, China; Emory University, UNITED STATES OF AMERICA

## Abstract

**Background:**

The “weekend effect”—inferior patient outcomes on weekends—has been reported across healthcare. Its impact on colonoscopy quality, particularly the adenoma detection rate (ADR), a key predictor of colorectal cancer risk, has not been fully established. This study investigated whether a weekend effect exists for ADR in a large cohort and analyzed potential contributing factors.

**Methods:**

This single-center retrospective study included consecutive patients undergoing colonoscopy from January 2022 to December 2023. Patients were categorized into Weekend (Saturday/Sunday) and Weekday (Monday-Friday) groups. The primary outcome was ADR (proportion with ≥1 adenoma). Data on demographics, withdrawal time, Boston Bowel Preparation Scale (BBPS), and endoscopist experience (Junior <5 years, Senior ≥5 years) were collected. Multivariable logistic regression identified factors independently associated with adenoma detection. Stratified analyses by patient subgroups, withdrawal time, and individual endoscopists were performed.

**Results:**

Of 16,550 colonoscopies (4,435 Weekend, 12,115 Weekday), ADR was significantly lower on weekends (24.1% vs. 26.2%, p = 0.006). Median withdrawal time was shorter on weekends (3.61 vs. 4.59 minutes, p < 0.001). After adjustment for confounders, weekend procedures were independently associated with lower ADR (aOR 0.892, 95% CI: 0.821–0.970, p = 0.007), indicating decreased odds of adenoma detection. Other independent predictors included female sex (aOR 0.613, p < 0.001), increasing patient age (aOR 1.050/year, p < 0.001), adequate bowel preparation (BBPS ≥6; aOR 1.407, p < 0.001), and procedure by a senior endoscopist (aOR 1.164, p < 0.001). Subgroup analyses revealed that the weekend effect was significant in specific patient groups compared to their weekday counterparts: females (aOR 0.868, p = 0.022), patients aged 50–59 years (aOR 0.698, p < 0.001), and those with inadequate preparation (BBPS<6; aOR 0.640, p = 0.010). The lower weekend ADR was primarily attributed to two junior endoscopists, with no significant effect observed among senior endoscopists.

**Conclusions:**

A significant weekend effect on ADR was confirmed, principally mediated by shorter withdrawal times and concentrated among junior endoscopists. The effect disproportionately affected women, middle-aged patients, and those with poor bowel preparation. Quality improvement efforts should therefore prioritize enforcing guideline-adherent withdrawal times, optimizing bowel preparation, and providing targeted support to less-experienced endoscopists during weekend services to standardize colonoscopy quality across all days of the week.

## Introduction

Colorectal cancer (CRC) remains a major global public health challenge due to its high incidence and mortality. According to the latest global cancer statistics, CRC ranks third worldwide, accounting for approximately 10.0% of all new cancer cases and 9.4% of cancer-related deaths [1,2]. The majority of CRCs develop from precancerous adenomatous polyps. Early detection and removal of these lesions through colonoscopy have been conclusively shown to reduce both CRC incidence and mortality [3,4]. However, the occurrence of interval colorectal cancer—cancer diagnosed after a negative screening colonoscopy—highlights the limitations of the procedure, primarily attributable to missed adenomas, suboptimal examination quality, and specific pathological features like serrated lesions.Adenoma detection rate (ADR), defined as the proportion of patients undergoing colonoscopy in whom at least one adenoma is detected, is internationally recognized as a core indicator for assessing the operational quality of endoscopists and the completeness of colonoscopy. Substantial research has confirmed an inverse correlation between an endoscopist’s ADR level and the patient’s risk of developing interval cancer after the examination [5], implying that a higher ADR is directly associated with better long-term patient outcomes. Therefore, identifying and controlling the various factors influencing ADR is crucial for enhancing the overall quality of colonoscopy and achieving effective prevention of colorectal cancer.Professional societies, such as the American Gastroenterological Association and the American Society for Gastrointestinal Endoscopy, recommend minimum ADR thresholds (e.g., ≥ 35% overall, or ≥30% for women and ≥40% for men in screening populations) to ensure effectiveness [6]. ADR is influenced by a multitude of factors, which can be categorized into patient-related (e.g., age, sex, bowel preparation quality) [7], procedure-related (e.g., cecal intubation rate, withdrawal time) [8], and endoscopist-related (e.g., experience, technique, fatigue) [9] domains.

The “weekend effect” in healthcare—the phenomenon where patients admitted or treated on weekends face a higher risk of adverse outcomes like mortality and complications compared to weekdays—has garnered increasing attention in recent years [10]. This pattern is theorized to arise from multiple contributing factors during weekends, such as reduced staffing with less experienced personnel, limited availability of ancillary services, variations in clinical workflows, and healthcare worker fatigue, all of which may influence procedural performance and diagnostic accuracy [11].

Evidence supporting the existence of a weekend effect has been reported across various medical specialties [12–16]. The most compelling evidence in endoscopy primarily stems from analyses of the UK National Endoscopy Database, which have consistently reported poorer quality indicators for colonoscopies performed on weekends. For instance, studies have found significantly lower mean number of polyps (MNP) and polyp detection rate (PDR)—a common surrogate for ADR—during weekend procedures, with adjusted relative risks and odds ratios of approximately 0.86–0.87 compared to weekdays, suggesting a 14% lower detection likelihood [17]. Supporting evidence also comes from other regions, such as a single-center study in China reporting a significantly lower weekend ADR (adjusted OR 0.78) [9]. Furthermore, although some studies reported non-significant trends (e.g., lower Saturday ADR or polyp detection), they align with the observed pattern [18].Some prior studies[8.14] have reported lower adenoma detection rates on Saturdays compared to weekdays. However, whether this difference represents a consistent temporal effect, its clinical significance, and—critically—the underlying procedural or operator-related mechanisms (such as variations in withdrawal time or endoscopist experience) remain poorly understood.

Given the critical role of the adenoma detection rate (ADR) as a key quality indicator for colonoscopy effectiveness, it is essential to investigate whether systematic variations in ADR occur depending on the day of the procedure. A rigorous examination of the weekend effect on ADR would not only clarify whether such disparities exist but could also help identify modifiable risk factors, thereby offering actionable targets for quality improvement initiatives aimed at standardizing high-quality care across all days of service. Therefore, the primary objective of this study was to examine the presence of a weekend effect on ADR in a large cohort of patients undergoing colonoscopy—including both screening and diagnostic procedures—and to analyze its potential influencing factors.

## Materials and methods

### Study design and data source

This single-center, retrospective study aimed to compare adenoma detection rates (ADR) between procedures performed on weekdays versus weekends. We extracted all colonoscopy data and corresponding histological reports (covering both screening and diagnostic indications) from the endoscopy and pathology databases of the Affiliated Hospital of Putian University. The inclusion period for the colonoscopy cases was from January 1, 2022, to December 31, 2023. Data management and statistical analysis were performed from October 1 to December 31, 2024.

### Ethical considerations

The study protocol was approved by the Ethics Committee of the Affiliated Hospital of Putian University (Approval No. 2024116) and registered in the Chinese Clinical Trial Registry (ChiCTR2400087977). The study was conducted in accordance with the principles of the Declaration of Helsinki. We followed the Strengthening the Reporting of Observational Studies in Epidemiology (STROBE) guidelines for reporting. To protect patient privacy, all data were fully de-identified prior to analysis. The authors had no access to any identifiable patient information during the research process.

### Study population

Inclusion criteria: All patients who underwent anesthesia-assisted colonoscopy at the endoscopy center, including both screening and diagnostic procedures.Exclusion criteria: 1) a diagnosis of colorectal cancer or a history of colectomy, 2) inflammatory bowel disease and subepithelial lesion or hereditary polyposis syndrome, 3) lack on information regarding polyp size and morphology of histologic findings

### Patient grouping and flow

**Fig 1:**
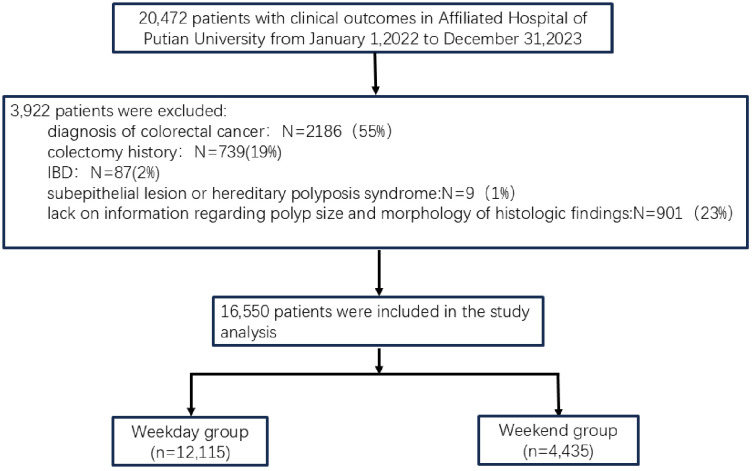
Flowchart depicting study grouping and patient inclusion process.

Patients were categorized into the Weekend Group if the colonoscopy was performed on a Saturday or Sunday, and the Weekday Group if performed from Monday to Friday. A complete flowchart detailing patient selection, exclusion, and final group allocation is provided in Fig 1.

### Endoscopic procedure

All colonoscopies were performed by a fixed cohort of full-time attending doctors, Critically, the same team of endoscopists performed procedures on both weekdays and weekends through a rotating schedule, with no use of part-time or temporary staff. For analysis, endoscopists were classified as Junior (<5 years of independent colonoscopy experience) or Senior (≥5 years). This 5-year threshold is commonly used in colonoscopy quality research to distinguish developing from established proficiency. Detailed characteristics, procedural volumes, and caseload distribution (weekday vs. weekend) for each endoscopist are provided in S1 Table.

### Variables and data collection

The following variables were systematically collected. Patient factors included age (as a continuous variable) and sex. Procedure-related factors encompassed the date (categorized as Weekend or Weekday), withdrawal time (minutes from cecal identification to scope removal), cecal intubation success, and bowel preparation quality. Bowel preparation was evaluated using the Boston Bowel Preparation Scale (BBPS), which assesses the cleanliness of the right, transverse, and left colon segments. Clinically, a ‘good’ preparation was defined as a total BBPS ≥ 6 with each segment scoring ≥ 2, while a ‘poor’ preparation was defined as any segment scoring < 2. For the purposes of statistical analysis, this was simplified into a dichotomous variable: ‘Adequate’ (total BBPS ≥ 6) versus ‘Inadequate’ (total BBPS < 6). Outcome factors focused on adenoma detection (yes/no) for calculating the Adenoma Detection Rate. For detected adenomas, characteristics including size, location, and morphology were recorded. Endoscopist experience level was documented as noted above.

### Statistical analysis

All statistical analyses were performed using SPSS software (version 25.0). Categorical variables are presented as frequencies and percentages and were compared using the Chi-square test or Fisher’s exact test, as appropriate. The normality of continuous variables was comprehensively assessed using the Kolmogorov–Smirnov test along with visual inspection of histograms and Q-Q plots. Normally distributed variables are expressed as mean ± standard deviation and compared using the independent samples t-test; non-normally distributed variables are expressed as median (interquartile range) and compared using the Mann–Whitney U test. A two-tailed p-value < 0.05 was considered statistically significant.

### Handling of missing data and procedure indication

There were no missing data for the key variables used in the primary analyses. Therefore, no imputation or exclusion for missingness was necessary.Additionally, while the distinction between screening and diagnostic colonoscopy is clinically pertinent, this information was not consistently recorded in our database with the reliability required for formal stratified analysis. Therefore, our primary analysis pools all eligible procedures to reflect real-world practice, and the potential implications of this are addressed in the Discussion.

### Multivariable and exploratory analyses

A multivariable binary logistic regression analysis was performed to identify factors independently associated with adenoma detection. Variables were considered for inclusion based on both clinical relevance and the results of univariate analyses (p < 0.05). The final model included procedure timing (weekend vs. weekday), patient sex, age, bowel preparation quality and endoscopist experience. Multicollinearity was assessed using the variance inflation factor (VIF), with a VIF < 5 considered to indicate no significant multicollinearity; no variable in the model exceeded this threshold. Results are reported as adjusted odds ratios (aOR) with 95% confidence intervals (CI).

We also performed stratified and exploratory analyses. Withdrawal time was categorized into quartiles for analysis of non-linear relationships. Subgroup analyses were conducted by patient demographics, bowel preparation quality, and individual endoscopist.

## Results

A total of 16,550 colonoscopy records were analyzed. Baseline characteristics and procedural outcomes are compared between weekday and weekend procedures in Table 1.

**Table 1:** Baseline characteristics and adenoma detection rate comparison between weekend and weekday colonoscopies.

	Weekend(n = 4435)	Weekday(n = 12115)	χ2/t/z	P value
Gender，n(%)			2.930	0.087
Male	2079(46.9)	5861(48.4)		
Female	2356(53.1)	6254(51.6)		
Age(years)	50.26 ± 13.43	50.42 ± 13.25	0.688	0.491
Withdrawal time(min)	3.61(2.79,4.83)	4.59(3.50,6.27)	30.995	<0.001
BBPS	6.85 ± 0.699	6.85 ± 0.675	−1.212	0.225
BBPS rank，n(%)			0.000	0.984
<6	298(6.7)	813(6.7)		
≥6	4137(93.3)	11302(93.3)		
Cecum Intubation, n (%)			/	0.565*
Success	4430(99.9)	12105(99.9)		
Failure	5(0.1)	10(0.1)		
ADR, n (%)	1069(24.1)	3173(26.2)	7.418	0.006

*Fisher’s Exact Test

Key procedural differences were observed. Colonoscopies performed on weekends had a significantly shorter median withdrawal time compared to those on weekdays (3.61 [IQR 2.79–4.83] min vs. 4.59 [3.50–6.27] min, p < .001) and a lower adenoma detection rate (24.1% vs. 26.2%, p = .006).

Patient demographics and other quality indicators were balanced. The groups were comparable in terms of gender (male: 48.4% vs. 46.9%; female: 51.6% vs. 53.1%, p = .087), mean age (50.26 ± 13.43 vs. 50.42 ± 13.25 years, p = .491), and bowel preparation quality, whether expressed as a total BBPS score (6.85 ± 0.699 vs. 6.85 ± 0.675, p = .225) or as a categorical variable (<6: 6.7% vs. 6.7%, p = .984). The cecal intubation rate was excellent and identical between groups (99.9% for both, p = .565 by Fisher’s exact test).

Table 2 compares patient and procedural characteristics between those with and without adenoma detection. Several factors were significantly associated with adenoma detection. Patients in whom an adenoma was detected were more likely to be male (56.1% vs. 45.2%, p < .001), older (mean age: 56.14 ± 11.36 vs. 48.39 ± 13.34 years, p < .001), and had a longer median withdrawal time (5.71 [IQR 4.40–7.85] vs. 3.94 [3.07–5.19] minutes, p < .001). The procedure was also more frequently performed on a weekday in the adenoma-detected group (74.8% vs. 72.7%, p = .006).

**Table 2:** Factors associated with adenoma detection in colonoscopy patients.

	Detected adenoma(n = 4242)	Non-detected adenoma(n = 12308)	χ2/t/z	P value
Gender，n(%)			151.924	<0.001
Male	2381(56.1)	5559(45.2)		
Female	1861(43.9)	6749(54.8)		
Age(years)	56.14 ± 11.36	48.39 ± 13.34	36.600	<0.001
Withdrawal time(min)，M(P25，P75)	5.71(4.40,7.85)	3.94(3.07,5.19)	47.501	<0.001
BBPS rank			3.104	0.078
<6	260(6.1)	851(6.9)		
≥6	3982(93.9)	11457(93.1)		
Cecum Intubation, n (%)			/	1.000*
Success	4238(99.9)	12297(99.9)		
Failure	4(0.1)	11(0.1)		
Time			7.418	0.006
Weekday	3173(74.8)	8942(72.7)		
Weekend	1069(25.2)	3366(27.3)		

*Fisher’s Exact Test

Conversely, no significant associations were found between adenoma detection and bowel preparation quality (BBPS ≥6: 93.9% vs. 93.1%, p = .078) or cecal intubation success rate (99.9% in both groups, p = 1.000 by Fisher’s exact test).

Table 3 compares the pathological characteristics of adenomas detected during weekday versus weekend procedures. No statistically significant differences were observed between the two groups across all assessed parameters. The anatomical distribution of adenomas—including the cecum, ascending colon, hepatic flexure, transverse colon, splenic flexure, descending colon, sigmoid colon, and rectum—was similar (p = 0.337). Likewise, the distributions by categorized size (≤5 mm, 6–9 mm, or ≥10 mm; p = 0.141) and by morphological shape (pedunculated, subpedunculated, or sessile; p = 0.375) did not differ significantly between weekdays and weekends.

**Table 3:** Characteristics of adenomas detected on weekday vs. weekend colonoscopies.

	Weekend(n = 1343)	Weekday(n = 3994)	χ2 value	P value
Adenoma location，n (%)			7.955	0.337
Cecum	77(5.7)	218(5.5)		
Ascending colon	234(17.4)	644(16.1)		
Hepatic flexure	115(8.6)	351(8.8)		
Transverse colon	282(21.0)	871(21.8)		
Splenic flexure	27(2.0)	66(1.7)		
Descending colon	120(8.9)	447(11.2)		
Sigmoid colon	283(21.1)	831(20.8)		
Rectum	205(15.3)	566(14.2)		
Classification of Adenoma size，n (%)			3.922	0.141
≤5	762(56.7)	2146(53.7)		
6-9	399(29.7)	1248(31.2)		
≥10	182(13.6)	600(15.0)		
Adenoma shape，n (%)			1.959	0.375
pedunculated	43(3.2)	121(3.0)		
Subpedunculated	692(51.5)	1977(49.5)		
sessile	608(45.3)	1896(47.5)		

Table 4 presents the results of univariable and multivariable binary logistic regression analyses for factors associated with adenoma detection.

**Table 4:** Univariable and multivariable logistic regression analysis of adenoma detection factors.

Factor	Univariable logistic regression analysis		Multivariable logistic regression analysis	
	OR(95%CI)	P value	OR(95%CI)	P value
Day of the week Weekday Weekend Gender	Reference 0.895(0.826-0.969)	0.006	0.892(0.821-0.970)	0.007
Male Female	Reference 0.644(0.600-0.691)	<0.001	0.613(0.570-0.660)	<0.001
Age BBPS rank	1.049(1.046-1.052)	<0.001	1.050(1.047-1.053)	<0.001
<6 ≥6	Reference 1.138(0.986-1.313)	0.078	1.407(1.209-1.638)	<0.001
Endoscopist experience				
Junior	Reference			
Senior	1.206(1.121-1.297)	<0.001	1.164(1.078-1.257)	<0.001

OR, odds ratio;CI, confidence interval.

In the univariable analysis, weekend procedures (vs. weekday: OR 0.895, 95%CI 0.826–0.969, p = .006), female sex (vs. male: OR 0.644, 95%CI 0.600–0.691, p < .001), increasing age (OR 1.049 per year, 95%CI 1.046–1.052, p < .001), and senior endoscopist experience (vs. junior: OR 1.206, 95%CI 1.121–1.297, p < .001) were significantly associated with adenoma detection. Bowel preparation quality (BBPS ≥6 vs. < 6: OR 1.138, 95%CI 0.986–1.313, p = .078) did not reach statistical significance in the univariable analysis.

The multivariable model, which included all the above variables, confirmed several independent associations. Weekend procedures (aOR 0.892, 95%CI 0.821–0.970, p = .007) and female sex (aOR 0.613, 95%CI 0.570–0.660, p < .001) remained independently associated with a lower likelihood of adenoma detection. Conversely, increasing age (aOR 1.050 per year, 95%CI 1.047–1.053, p < .001), adequate bowel preparation (BBPS ≥6 vs. < 6: aOR 1.407, 95%CI 1.209–1.638, p < .001), and senior endoscopist experience (aOR 1.164, 95%CI 1.078–1.257, p < .001) were independent predictors of a higher adenoma detection rate.

Table 5 presents the results of stratified analyses examining the “weekend effect” on ADR across different patient subgroups (gender, age, and BBPS category), with ORs adjusted for gender, age, endoscopist experience, and BBPS rank.

**Table 5:** Stratified analysis of adenoma detection rates by gender, age, and bowel preparation quality.

Factor		Time	Adenoma detection	χ2 value	P value	Weekend vs weekday OR(95%CI)*	P value
Gender	Male	Weekday	1783/5861(30.4)	2.008	0.156	Reference	
		Weekend	598/2079(28.8)			0.914(0.815-1.025)	0.125
	Female	Weekday	1390/6254(22.2)	5.402	0.025	Reference	
		Weekend	471/2356(20.0)			0.868(0.769-0.980)	0.022
Age(years)	<20	Weekday	1/95(1.1)		0.514**	Reference	
		Weekend	1/41(2.4)			5.767(0.193-159.881)	0.312
	20 ~ 29	Weekday	35/579(6.0)	0.888	0.346	Reference	
		Weekend	17/215(7.9)			1.369(0.748-2.507)	0.315
	30 ~ 39	Weekday	248/2260(11.0)	0.323	0.570	Reference	
		Weekend	101/864(11.7)			1.065(0.832-1.363)	0.576
	40 ~ 49	Weekday	545/2438(22.4)	4.547	0.033	Reference	
		Weekend	182/957(19.0)			0.832(0.689-1.005)	0.059
	50 ~ 59	Weekday	1105/3593(30.8)	18.056	<0.001	Reference	
		Weekend	256/1066(24.0)			0.698(0.595-0.818)	<0.001
	60 ~ 69	Weekday	889/2374(37.4)	0.056	0.812	Reference	
		Weekend	389/1027(037.9			1.030(0.884-1.199)	0.706
	≥70	Weekday	350/776(45.1)	0.137	0.711	Reference	
		Weekend	123/265(46.4)			1.058(0.799-1.400)	0.695
BBPS rank	<6	Weekday	204/813(25.1)	4.829	0.028	Reference	
		Weekend	56/298(18.8)			0.640(0.454-0.901)	0.010
	≥6	Weekday	2969/11302(26.3)	5.032	0.025	Reference	
		Weekend	1013/4137(24.5)			0.913(0.838-0.995)	0.038

Note: This table presents the comparison of Adenoma Detection Rates (ADR) between weekends and weekdays across different patient subgroups. The analyzed factors include: 1) Patient sex (Male vs. Female); 2) Patient age (grouped: < 20, 20–29, 30–39, 40–49, 50–59, 60–69, ≥ 70 years); 3) Bowel preparation quality (Total Boston Bowel Preparation Scale score <6 vs. ≥ 6). Odds ratios (OR) and 95% confidence intervals (CI) were adjusted for sex, age, endoscopist experience, and BBPS score.CI, confi dence interval; *OR, odds ratio

**Fisher’s Exact Test

Gender-stratified analysis revealed a sex-specific pattern. While no significant weekend-weekday difference was observed in male patients (aOR 0.914, 95% CI: 0.815–1.025, p = 0.125), female patients had a significantly lower ADR on weekends (aOR 0.868, 95% CI: 0.769–0.980, p = 0.022).

Age-stratified analysis identified the 50–59 years age group as particularly susceptible, showing a pronounced reduction in weekend ADR (aOR 0.698, 95% CI: 0.595–0.818, p < 0.001). A trend towards a lower weekend ADR was noted in the 40–49 years group (aOR 0.832, 95% CI: 0.689–1.005, p = 0.059). No statistically significant weekend-weekday differences were observed in other age strata (all p > 0.05): < 20 years (aOR 5.767, 95% CI: 0.193–159.881, p = 0.312), 20–29 years (aOR 1.369, 95% CI: 0.748–2.507, p = 0.315), 30–39 years (aOR 1.065, 95% CI: 0.832–1.363, p = 0.576), 60–69 years (aOR 1.030, 95% CI: 0.884–1.199, p = 0.706), and ≥70 years (aOR 1.058, 95% CI: 0.799–1.400, p = 0.695).

Analysis stratified by bowel preparation quality showed that the weekend effect was most pronounced and statistically significant among patients with inadequate preparation (BBPS < 6; aOR 0.640, 95% CI: 0.454–0.901, p = 0.010). Among patients with adequate preparation (BBPS ≥ 6), the weekend ADR was also significantly lower, though the effect size was modest (aOR 0.913, 95% CI: 0.838–0.995, p = 0.038).

Stratified analyses were conducted to further investigate the “weekend effect” by withdrawal time and individual endoscopist (Table 6). Withdrawal time was categorized into quartiles (Q1 = 3.27, Q2 = 4.32, Q3 = 5.87 minutes) to explore non-linear relationships. Notably, within most withdrawal time strata, a reverse relationship was observed: when withdrawal time was longer, weekend ADR became significantly higher than weekday ADR. Compared to weekdays, weekend procedures had a higher ADR in the 4.32-minute stratum (aOR 1.765, 95% CI: 1.401–2.002, p < .001), the 5.87-minute stratum (aOR 1.471, 95% CI: 1.253–1.727, p < .001), and the > 5.87-minute stratum (aOR 1.235, 95% CI: 1.034–1.474, p = .012). No significant difference was found in the shortest stratum (≤3.27 minutes: aOR 1.198, 95% CI: 0.942–1.524, p = .141).

**Table 6:** Factors associated with adenoma detection: stratified analysis by withdrawal time and individual endoscopists.

Factor		Time	Adenoma detection	χ2 value	P value	Weekend vs weekday OR(95%CI)*	P value
Withdrawal time (min)	≤3.27	Weekday	158/2370(6.7)	2.066	0.157	Reference	
		Weekend	138/1766(7.8)			1.198(0.942-1.524)	0.141
	~4.32	Weekday	419/2966(14.1)	40.249	<0.001	Reference	
		Weekend	261/1174(22.2)			1.765(1.401-2.002)	<0.001
	~5.87	Weekday	898/3250(27.6)	33.985	<0.001	Reference	
		Weekend	334/885(37.7)			1.471(1.253-1.727)	<0.001
	>5.87	Weekday	1698/3529(48.1)	10.099	0.001	Reference	
		Weekend	336/610(55.1)			1.235(1.034-1.474)	0.012
Endoscopist	Experience (years)						
1	1	Weekday	330/1210(27.3)	9.562	0.002	Reference	
		Weekend	100/497(20.1)			0.682(0.525-0.887)	0.004
2	2	Weekday	379/1618(23.4)	3.709	0.054	Reference	
		Weekend	83/435(19.1)			0.724(0.548-0.955)	0.022
3	3	Weekday	447/1863(24.0)	4.238	0.040	Reference	
		Weekend	123/616(20.0)			0.804(0.636-1.016)	0.068
4	5	Weekday	313/1210(25.9)	0.543	0.461	Reference	
		Weekend	152/552(27.5)			1.097(0.866-1.390)	0.441
5	6	Weekday	381/1319(28.9)	0.229	0.632	Reference	
		Weekend	183/657(27.9)			0.962(0.775-1.195)	0.727
6	7	Weekday	497/1872(26.5)	1.316	0.251	Reference	
		Weekend	170/699(24.3)			0.884(0.716-1.092)	0.253
7	8	Weekday	430/1596(26.9)	0.295	0.587	Reference	
		Weekend	160/620(25.8)			0.911(0.731-1.135)	0.405
8	15	Weekday	396/1427(27.8)	0.029	0.864	Reference	
		Weekend	98/359(27.3)			1.007(0.770-1.317)	0.958

Note: This table presents adenoma detection stratified by withdrawal time quartiles and individual endoscopists. The analyzed factors include: 1) Withdrawal time (categorized into four strata based on the 25th, 50th, and 75th percentiles of the total population: ≤ 3.27 min, 3.28–4.32 min, 4.33–5.87 min, > 5.87 min); 2) Individual endoscopist (numbered 1–8, with years of independent colonoscopy experience indicated). *Odds ratios (OR) and 95% confidence intervals (CI) were adjusted for sex, age, endoscopist experience, and BBPS score.

Analysis by individual endoscopist (see Supplementary Table S1 for details) revealed that the overall weekend decline in ADR was predominantly attributable to two junior endoscopists (Endoscopist 1: aOR 0.682, 95% CI: 0.525–0.887, p = .004; Endoscopist 2: aOR 0.724, 95% CI: 0.548–0.955, p = .022). A non-significant trend was observed for another junior endoscopist (Endoscopist 3: aOR 0.804, 95% CI: 0.636–1.016, p = .068). In contrast, none of the five senior endoscopists showed a statistically significant weekday-weekend difference in ADR (all p > .05). Importantly, the caseload distribution between weekdays and weekends was comparable across all endoscopists (Supplementary Table S1).

## Discussion

In this study, we retrospectively analyzed 16,550 colonoscopy procedures and confirmed the presence of a “weekend effect” in adenoma detection rates (ADR), with a significant 2% absolute reduction on weekends. A corresponding shortening of median withdrawal time (3.61 vs. 4.59 minutes) was also observed. Given comparable baseline patient characteristics, a key question is whether the ADR deficit is attributable to shorter withdrawal time or other factors.

To further analyze the reasons for the ADR difference, we examined the characteristics of adenomas detected on both days. Previous studies have reported that adenoma size, location, and morphology affect detection rates [22]. However, in our study, no significant differences were observed in these pathological characteristics between weekends and weekdays. This suggests that the spectrum of lesions detected is similar and that the “weekend effect” is not driven by inherent differences in the adenomas themselves.The observed 2% absolute reduction, while modest, has tangible implications. Extrapolating from our annual procedural volume, this difference could translate to approximately 160 additional adenomas being missed each year at our center if the pattern persists. Given the established link between higher ADR and lower interval colorectal cancer risk—where even a 1% ADR increase is associated with a 3% decrease in cancer risk [23]—a persistent 2% deficit could attenuate colonoscopy’s long-term protective effect at a population level.

Regarding quality benchmarks, our cohort’s overall ADR (25.63%, a weighted average) meets and exceeds the minimum national benchmark for colonoscopy quality in China (≥20%) and aligns with gender-specific targets (male >25%, female >15%). While more stringent international guidelines (e.g., from ACG/ASGE) propose targets of ≥35% for screening populations [6], our findings are appropriately contextualized within the prevailing national standards. The consistent weekend decline to 24.1%—though still above the national minimum—represents a significant internal “performance gap” relative to our own higher weekday standard (26.2%). This underscores that continuous quality monitoring and improvement are imperative.

We propose that the weekend effect on ADR arises from the convergence of several interrelated factors spanning procedural, operator, and patient domains.

Withdrawal time is a well-established, critical factor influencing ADR. International consensus guidelines, based on pivotal studies, recommend a minimum mean withdrawal time of 6 minutes for screening colonoscopies, as times below this threshold are associated with significantly lower ADR [24]. Beyond this minimum, a dose-response relationship exists, with evidence suggesting that extending withdrawal time to 8 minutes or longer can further optimize adenoma detection [6,25,26]. These specific thresholds (6 and 8 minutes) have been historically studied as pragmatic, data-driven benchmarks.In our study, withdrawal time was significantly shorter during weekend procedures (median: 3.61 vs. 4.59 minutes on weekdays, p < 0.001). Notably, both median values fell below the guideline-recommended 6-minute benchmark, indicating a systemic opportunity for improvement that was more pronounced on weekends. The reasons for this baseline shortfall were not the primary focus of our study, but such findings may reflect operational realities common in high-volume endoscopic units, where system-level constraints can influence procedural pacing. Crucially, our data demonstrate that within this operational context, withdrawal time undergoes a further significant compression on weekends.This finding is further informed by examining the distribution. The substantial overlap in interquartile ranges (IQRs: weekend 2.79–4.83 min; weekday 3.50–6.27 min) indicates that while the central tendency was shorter on weekends, there was considerable variability within both groups. This suggests the “weekend effect” is not due to a uniform, rigid time cut-off but reflects a shift in the distribution—a higher proportion of procedures clustering in the shorter duration range on weekends. This pattern implicates factors influencing procedural pace or circumstance rather than a simple mandate to finish quickly.Our stratified analysis revealed a consistent, positive relationship between longer withdrawal time and higher ADR. A seemingly paradoxical finding was that within each withdrawal time stratum, weekend ADR was often numerically higher, yet the overall unstratified ADR was lower. We hypothesize this discrepancy stems from a disproportionate distribution of cases: a much larger proportion of weekend procedures fell into the shortest withdrawal time quartile (≤3.27 minutes), where ADR is inherently lowest. This distributional shift effectively “diluted” the overall weekend ADR.Therefore, the key issue is not a difference in ADR at a given withdrawal time, but a systematic shortening of withdrawal time on weekends, which places a greater volume of procedures into the low-ADR, short-duration category. A direct recommendation is to focus on achieving and sustaining guideline-adherent withdrawal times, particularly during weekend lists.

Subgroup analyses revealed a significantly lower weekend ADR among females. While female anatomy (e.g., increased bowel tortuosity, history of gynecological surgery) poses a constant technical challenge [27,28], it does not alone explain a weekend-specific decline. We hypothesize that the weekend procedural environment (e.g., faster pacing, operator fatigue) may disproportionately compromise the extra time and meticulousness required for optimal inspection in anatomically challenging colons, thus amplifying the impact of these constant difficulties.

Patients aged 50–59 also had a significantly lower weekend ADR. We hypothesize that patients in middle adulthood may experience stressors affecting bowel preparation. An alternative or complementary explanation is that the generally lower adenoma prevalence in younger adults may inadvertently reduce endoscopist vigilance, an effect potentially magnified during weekend procedures. These non-mutually exclusive hypotheses require further research.

The critical influence of bowel preparation quality on colonoscopy outcomes is well-established [6,19–21]. Our analysis, which adjusted for key confounders including endoscopist experience, clarifies its role as a potent modifier of the weekend effect (Table 5). We observed a pronounced gradient in the magnitude of the weekend effect dependent on preparation quality. The most severe impact was among patients with inadequate bowel preparation (BBPS < 6), where weekend procedures were associated with a substantially lower adenoma detection rate (aOR 0.640, 95% CI: 0.454–0.901, p = 0.010). Crucially, even among patients with adequate preparation (BBPS ≥ 6), a statistically significant weekend decline persisted, albeit with a markedly attenuated effect size (aOR 0.913, 95% CI: 0.838–0.995, p = 0.038).

This gradient reveals important mechanistic insights. Firstly, a poorly cleansed colon creates a suboptimal baseline where mucosal visualization is inherently compromised. In this setting, weekend-associated factors—such as subtle time constraints, cumulative fatigue, or variations in operator diligence—can catastrophically amplify the consequence of any lapse in meticulous inspection or debris clearance. The result is a dramatically increased risk of missed adenomas. Secondly, the persistence of a small but significant effect even with adequate preparation suggests that weekend factors may influence aspects of performance beyond what is rectified by a clear visual field. This could include subtle reductions in cognitive vigilance for subtle, flat lesions, or pattern recognition efficiency. However, the strong attenuation of the effect by excellent preparation underscores its fundamental role as a critical buffer against weekend-related quality erosion. It establishes a resilient technical foundation that minimizes the operational “noise” through which other weekend variables can exert an effect.

Therefore, our findings highlight a clear practical priority: Aggressively optimizing bowel preparation is a highly effective and actionable strategy to mitigate the weekend effect, particularly for neutralizing its most severe manifestations observed under suboptimal conditions. Concurrently, the residual small effect in well-prepared patients signals that achieving truly uniform, high-quality performance across all days of the week may require complementary measures addressing other potential contributors, such as operator factors and workflow.

Endoscopist proficiency is crucial for adenoma detection [29,30]. In our study, the weekend effect was isolated to the two most junior endoscopists (<5 years’ experience), while senior endoscopists (≥5 years) maintained consistent performance. This was not due to imbalanced workload. We hypothesize that less experienced operators lack the automated patterns and cognitive resilience of their senior colleagues, making them more susceptible to degradation under weekend stressors.Cumulative provider fatigue and potential workload variation are key, though difficult-to-quantify, factors [31–33]. Fatigue after a workweek, potentially exacerbated by weekend scheduling, may impair vigilance and technique, disproportionately affecting junior staff. The absence of formal intra-procedural senior support (a constant condition) further underscores that performance gaps are borne by the individual operator. These factors should be acknowledged as important potential confounders.

The weekend effect on ADR likely arises from the convergence of a systemic tendency toward shorter withdrawal times with a clinical environment that amplifies the impact of this haste, particularly on less experienced operators and in specific patient subgroups. Comparable cecal intubation rates confirm the deficit arises during the withdrawal phase, not from incomplete examinations.

### Implications for practice

Our analysis elucidates mechanisms that point toward stratified strategies for mitigating the weekend disparity in colonoscopy quality. The findings indicate that the “weekend effect” is not uniform but is significantly mediated by modifiable procedural and operator factors.

First, withdrawal time management emerges as a critical and immediate lever. Our data reveal a counterintuitive interaction: within longer withdrawal time strata, weekend performance matches or exceeds weekday levels. This suggests that the overall deficit is largely attributable to a systemic shortening of withdrawal time on weekends, not an inherent inability to detect adenomas within a given time frame. Therefore, a primary intervention should be the enforcement and monitoring of guideline-adherent withdrawal times, with particular vigilance during weekend schedules to counteract this tendency.

Second, excellent bowel preparation is a powerful, patient-level buffer. Our stratified analysis confirms that while the weekend effect persists even with adequate preparation, its magnitude is drastically amplified by poor preparation. Aggressively optimizing bowel preparation is therefore a foundational strategy, especially for weekend procedures, to eliminate a major preventable contributor to the performance gap.

Third, targeted support for less experienced endoscopists is warranted. The concentration of the weekend effect among junior operators, independent of caseload distribution, highlights a vulnerability. Interventions such as structured mentoring, fatigue-aware scheduling, and real-time feedback could help bridge this experience-related performance gap on weekends.

Finally, a systems-based approach is beneficial. Scheduling higher-risk patients (e.g., females, patients aged 50–59, or those with anticipated preparation challenges) on weekdays when feasible can be a pragmatic risk-mitigation tactic. Additionally, optimizing weekend staffing models to ensure a balanced mix of experience and fostering a culture of continuous skill enhancement for all endoscopists are fundamental to sustaining high-quality performance across the entire week.

### Limitations

This study has several limitations. First, its single-center, retrospective design limits generalizability and precludes causal inference. Second, we could not adjust for unmeasured confounders such as detailed comorbidities, prior colonoscopy history, or, critically, direct measures of provider fatigue and intra-shift workload variation, which may meaningfully influence ADR. Third, the inability to stratify by procedure indication (screening vs. diagnostic) due to inconsistent data recording prevents assessment of case-mix differences. Fourth, the inherent imbalance in exposure time (five weekdays vs. two weekend days) may introduce subtle temporal biases. While baseline characteristics were balanced and models adjusted for available confounders, these limitations underscore the need for prospective validation.

### Conclusions

This study confirms a significant “weekend effect” on adenoma detection rates (ADR) in a large, single-center cohort after adjusting for established patient- and procedure-related factors. We found this effect is primarily driven by a systematic shortening of withdrawal time on weekends and is concentrated among junior endoscopists. These findings elucidate the key mechanisms behind the ADR disparity and highlight that internal temporal variations can reveal specific quality gaps, even when overall institutional performance meets established benchmarks.

## Supporting information

S1 Table Characteristics and procedure distribution of endoscopists involved in the study. (DOCX)

## Abbreviations

ADR: adenoma detection rate
BBPS: Boston bowel preparation scale
CIR: cecal intubation rate
CRC: colorectal cancer
