# Supplementary material for: Is there a “weekend effect” in adenoma detection rate?A single-center retrospective study
Source: PLoS One. 2026 Apr 6;21(4):e0345613. doi: 10.1371/journal.pone.0345613 (PMC13052870; doi:10.1371/journal.pone.0345613)
Supplement: S1 Table — (DOCX) [file pone.0345613.s001.docx]

**Supplementary Table S1:Characteristics and procedure distribution of endoscopists involved in the study.**

| Endoscopist No. | Experience (years) | Level (Junior/Senior) | Total Procedures (n) | Weekend Procedures (n) | Weekend Proportion (%) | Weekday ADR (%) | Weekend ADR (%) |
| --- | --- | --- | --- | --- | --- | --- | --- |
| 1 | 1 | Junior | 1707 | 497 | 29.1 | 27.3 | 20.1 |
| 2 | 2 | Junior | 2053 | 435 | 21.2 | 23.4 | 19.1 |
| 3 | 3 | Junior | 2479 | 616 | 24.8 | 24.0 | 20.0 |
| 4 | 5 | Senior | 1762 | 552 | 31.3 | 25.9 | 27.5 |
| 5 | 6 | Senior | 1976 | 657 | 33.2 | 28.9 | 27.9 |
| 6 | 7 | Senior | 2571 | 699 | 27.2 | 26.5 | 24.3 |
| 7 | 8 | Senior | 2216 | 620 | 28.0 | 26.9 | 25.8 |
| 8 | 15 | Senior | 1786 | 359 | 20.1 | 27.8 | 27.3 |
| Total/Average | - | - | 16550 | 4435 | 26.8 | 26.2 | 24.1 |

- Level: Endoscopists were classified as Junior if they had <5 years of independent colonoscopy experience, and Senior if they had ≥5 years of experience.
- Weekend Proportion (%) = (Weekend Procedures / Total Procedures) × 100%.
- Weekday and Weekend ADR were calculated based on adenoma detection per endoscopist as shown in the main manuscript (Table 6).
- The Total/Average row shows the aggregate data for the entire cohort. The "Weekend Proportion" is the overall percentage of weekend procedures (4,435/16,550), and the ADR values are the overall rates from Table 1.
